# So close: remaining challenges to eradicating polio

**DOI:** 10.1186/s12916-016-0594-6

**Published:** 2016-03-14

**Authors:** Michael J. Toole

**Affiliations:** Burnet Institute, 85 Commercial Rd, Melbourne, 3004 Australia

**Keywords:** Elimination, Eradication, Polio, Poliomyelitis, Poliovirus

## Abstract

The Global Polio Eradication Initiative, launched in 1988, is close to achieving its goal. In 2015, reported cases of wild poliovirus were limited to just two countries – Afghanistan and Pakistan. Africa has been polio-free for more than 18 months. Remaining barriers to global eradication include insecurity in areas such as Northwest Pakistan and Eastern and Southern Afghanistan, where polio cases continue to be reported. Hostility to vaccination is either based on extreme ideologies, such as in Pakistan, vaccination fatigue by parents whose children have received more than 15 doses, and misunderstandings about the vaccine’s safety and effectiveness such as in Ukraine. A further challenge is continued circulation of vaccine-derived poliovirus in populations with low immunity, with 28 cases reported in 2015 in countries as diverse as Madagascar, Ukraine, Laos, and Myanmar. This paper summarizes the current epidemiology of wild and vaccine-derived poliovirus, and describes the remaining challenges to eradication and innovative approaches being taken to overcome them.

## Background

We may be witnessing the last stand of the wild poliovirus (WPV). In 2015, approximately 70 cases of WPV were reported in only two countries – Pakistan and Afghanistan, representing approximately 0.02 % of the global polio burden in 1988, the year the Global Polio Eradication Initiative (GPEI) was launched [1]. As of the end of February, only five cases had been reported in 2016, and all in Pakistan [2]. Polio is not a ‘tropical disease’ and therefore represented a major problem in many developed countries until the 1960s. For example, in 1952, there were 58,000 cases of paralytic polio in the United States, resulting in 3,145 deaths and 22,000 people left with varying degrees of paralysis [3].

WPV is transmitted via the fecal-oral route and, therefore, thrives in environments where sanitation is poor. There are three types of WPV – 1, 2, and 3 – all of which can cause severe paralysis or death. However, for every 200 people infected (mostly children), only one develops paralysis, 20 have non-specific symptoms, and 180 are asymptomatic [4]. This creates challenges for surveillance as the virus may circulate undetected. There has been no case of WPV2 since October 1999 and it was officially declared eradicated in September 2015; WPV3 has not been detected since November 2012.

The most common polio vaccine used worldwide is the trivalent oral polio vaccine (OPV), which contains three types of live, attenuated (weakened) vaccine-viruses. OPV induces immunity against all three types of WPV in the gut of a vaccinated child, thus preventing the viruses from entering the bloodstream and the nervous system to cause paralysis. In most countries, three or four vaccine doses are adequate to protect a child from infection. However, in areas where water quality, sanitation, and hygiene are poor, children may require ten or more doses because the many other viruses and bacteria that infect their gut interfere with the body’s ability to mount an effective immune response, as was the case in the last reservoirs of polio in India – impoverished and densely populated districts of Bihar and Uttar Pradesh States [5]. Therefore, vaccines that target just one (‘monovalent’) or two (‘bivalent’) of the virus strains and induce higher levels of immunity than the trivalent vaccine were developed [6].

Vaccine-associated paralytic poliomyelitis occurs in an estimated 1 in 2.7 million children receiving their first OPV dose, which is why most upper-income countries have switched to the injectable, inactivated (dead) polio vaccine, which induces immunity in the bloodstream. Additionally, after a child is vaccinated, some of the vaccine-virus may be genetically altered during replication upon excretion, and is subsequently termed a vaccine-derived poliovirus. On rare occasions, there are enough susceptible children for the excreted viruses to begin circulating and cause paralytic cases in populations with low immunity; these viruses are called circulating vaccine-derived polioviruses (cVDPV) [7].

## Global Polio Eradication Initiative (GPEI)

The GPEI was launched in 1988, when approximately 350,000 children were paralyzed by polio every year [8]. Initial progress was rapid; the last case of WPV in the WHO region of the Americas occurred in 1993 and the last case in the Western Pacific region (which includes China) occurred in 1997 [9]. However, the initial target of the GPEI to eradicate polio from the world by the year 2000 was not achieved [10]. Progress was slow for the subsequent decade; the number of cases reported in 2010 was higher than in 2000 and 20 countries were still reporting polio.

Since 2010, although deadlines for interrupting WPV transmission were missed in 2012 and 2015, significant progress has been made, including elimination in India and Nigeria. Globally, WPV cases declined from 1,352 in 2010 to 74 in just two countries in 2015 (WHO data 2 March 2016) [11]. The African continent has been WPV-free since August 2014.

**Fig. 1:**
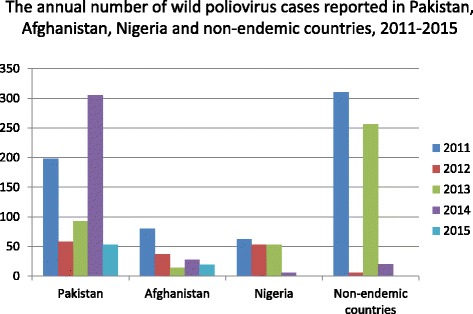
The annual number of wild poliovirus cases reported in Pakistan, Afghanistan, Nigeria and non-endemic countries, 2011-2015. Source: World Health Organization. Global Polio Eradication Initiative. Data and Monitoring. http://www.polioeradication.org/Dataandmonitoring.aspx

Figure 1 shows the number of reported cases of WPV in Afghanistan, Pakistan, Nigeria, and non-endemic countries between 2011 and 2015 (data from [2]).

The GPEI is led by five partners that comprise the Polio Oversight Board, namely the World Health Organization, UNICEF, Rotary International, the US Centers for Disease Control and Prevention, and the Bill & Melinda Gates Foundation. The Independent Monitoring Board (IMB) was convened in late 2010 at the request of the World Health Assembly to monitor and guide progress towards eradication.

We are on the cusp of a polio-free world; however, several challenges need to be overcome in order to meet the deadline set by the Polio Oversight Board to interrupt WPV transmission in 2016. This commentary will discuss these major barriers, and briefly illustrate the paths through which these issues could be overcome.

## Major challenges

### Insecurity

Since 2010, armed conflict and insecurity have been major constraints to accessing children for polio vaccination in the endemic countries of Afghanistan, Pakistan and Nigeria, and countries that experienced outbreaks such as Somalia, Cote d’Ivoire, Syria, Democratic Republic of Congo, Mali, and Iraq.

Nevertheless, innovative communication and access strategies, such as employing local respected intermediaries to negotiate with anti-government elements, have overcome the barrier of insecurity in most countries. In Northeast Nigeria, where the militant Boko Haram group disrupted vaccination campaigns, temporary ‘health camps’ were established offering a range of health services in addition to the polio vaccine [12]. Visiting Afghanistan on behalf of the IMB, I witnessed several innovative efforts to overcome security barriers. In Kandahar province, permanent polio vaccination teams were established to take advantage of all opportunities to vaccinate children such as market days. In Jalalabad, ‘women’s courtyards’ provided safe spaces for women to discuss the benefits of vaccination.

### Hostility to vaccination

The refusal by parents to vaccinate their children is due to a number of factors, ranging from ‘vaccination fatigue’, in countries where children may have been vaccinated 15 or more times, to outright bans by militant groups such as Al Shabaab in Somalia [13]. The situation is most toxic in Pakistan, where the Taliban has banned polio vaccination in North and South Waziristan and incited violence against vaccinators and their police escorts [14]. As recently as January 13, 2016, a suicide bomber detonated explosives outside a polio vaccination center in the city of Quetta, killing 15 and wounding 24 people [15].

A number of initiatives have been launched to counter misinformation about polio vaccine in the guise of religious doctrine. A meeting of Islamic scholars convened at Cairo’s renowned Al-Azhar University in March 2014, and led to the formation of an Islamic Advisory Group for Polio Eradication [16]. Fatwas supporting polio vaccination by the Grand Imam of Al Azhar and other leading Islamic clerics have since been widely disseminated and tens of thousands of social mobilizers have been trained to disseminate information on vaccination by word-of-mouth. During a visit to Chad on behalf of the IMB, I visited a community where the spiritual leader, Sheikh Ousman, preached that it was every parent’s duty to vaccinate their children against polio and other preventable diseases. As a result, according to local Chadian officials (unpublished data), a 100 % coverage in vaccination campaigns was achieved.

### Vaccine-derived polio

As described earlier, vaccine-derived poliovirus may sometimes circulate, causing paralytic cases in populations with low immunity. Prior to 2015, most cases of cVDPV had been Type 2. Since there has been no case of WPV Type 2 since 1999, WHO’s Strategic Advisory Group of Experts on Immunization (SAGE) concluded, in October 2015, that the switch from trivalent to bivalent OPV (Types 1 and 3) should go ahead globally in April 2016 [17]. There was a resurgence of Type 1 cVDPV in 2015, with 17 cases reported in Laos, Madagascar, and Ukraine, reflecting diminished levels of immunity in these populations; for example, in mid-2015, only half of Ukrainian children had been fully vaccinated against polio [18].

## The way forward

The title of the IMB’s most recent report was “Now is the Time for Peak Performance” [19]. Indeed, the current low season of transmission (through May 2016) is the time to implement high-quality campaigns that achieve the greatest possible vaccination coverage, especially in areas such as southern Afghanistan, where large proportions of children are missed during campaigns.

The combined use of OPV and inactivated polio vaccine in campaigns is likely to boost population immunity and should be employed routinely in endemic countries and others that are vulnerable to reintroduction of WPV (e.g. Nigeria, Cameroon, Chad, Somalia, and Yemen).

Environmental surveillance should be rapidly expanded throughout Pakistan and Afghanistan coupled with rigorous outbreak response action when positive samples are found. Since WPV causes paralysis in less than 1 % of infected children, an ‘orphan’ virus may circulate undetected by routine acute flaccid paralysis surveillance [20]. In addition, surveillance ‘blackspots’ need to be addressed in countries where international surveillance indicators are not met.

High-level political commitment is essential at the national level to ensure accountability for campaign quality by provincial governors in Afghanistan and state governments in Pakistan and Nigeria. However, at the frontline, the polio campaign needs to be politically neutral to avoid negative responses from anti-government elements.

The rest of the world cannot be complacent. Reintroduction of WPV led to a major outbreak in Somalia in 2013 after a ban by armed militants led to half a million children going unimmunized for three years. Recent clusters of vaccine-derived poliovirus in countries as diverse as Ukraine, Myanmar, and Madagascar indicate low population immunity.

## Conclusions

If we are to succeed in eradicating the second human disease in history during 2016, an unprecedented global endeavor will be required, involving parents, vaccinators, community and religious leaders, state, province and national governments, political leaders, international technical agencies, and the world’s major aid donors.

While eradication of this terrible disease is clearly for the global public good, we cannot presume that support will be universal. Radical political forces in a number of countries have demonstrated that they will put their own agenda ahead of the well-being of children. Innovative (and politically-neutral) actions are needed to overcome the barriers of insecurity and hostility.

The GPEI provides important lessons for future disease eradication efforts. First, disease eradication is not easy and requires sustained financial and technical support. Second, it will not succeed without high-level political commitment, intense coordination, and mobilization of communities and their leaders. Third, global campaigns can benefit from oversight by an independent review body such as the Polio IMB. This time next year, let us hope that the fourth lesson will be that it can be done.

## References

[1] US Centers for Disease Control and Prevention. Infographic: The Time to Eradicate Polio is Now. http://www.cdc.gov/globalhealth/immunization/infographic/eradicate_polio.htm. Accessed 16 January 2016.

[2] World Health Organization. Global Polio Eradication Initiative. Data and Monitoring. http://www.polioeradication.org/Dataandmonitoring.aspx. Accessed 29 February 2016.

[3] Smith JS (1990). Patenting the Sun: Polio and the Salk Vaccine.

[4] US Centers for Disease Control and Prevention. Global Health – Polio. http://www.cdc.gov/polio/about/. Accessed 27 February 2016.

[5] United Nations Children’s Fund (UNICEF) (2003). A critical leap to polio eradication in India.

[6] Sutter RW, John TJ, Jain H, Agarkhedkar S, Ramanan PV, Verma H (2010). Immunogenicity of bivalent types 1 and 3 oral poliovirus vaccine: a randomised, double-blind, controlled trial. Lancet.

[7] Kew OM, Wright PF, Agol VI, Delpeyroux F, Shimizu H, Nathanson N (2004). Circulating vaccine-derived polioviruses: current state of knowledge. Bull World Health Organ.

[8] Polio: eradication. Nature Web Focus. http://www.nature.com/nature/focus/polio/eradication.html. Accessed March 8, 2016.

[9] World Health Organization. Global Polio Eradication Initiative. History of polio. http://www.polioeradication.org/Polioandprevention/Historyofpolio.aspx. Accessed 28 February 2016.

[10] Independent Monitoring Board of the Global Polio Eradication Initiative. Report, April 2011. http://www.polioeradication.org/Portals/0/Document/Data&Monitoring/IMB_Reports/IMB_Report_April2011.pdf.

[11] World Health Organization. Global Polio Eradication Initiative. Weekly Polio Update. http://www.polioeradication.org/dataandmonitoring/poliothisweek.aspx. Accessed 8 March 2016.

[12] Patil N. Health Camps: Expanding Access to Healthcare during Polio Immunization Campaigns in Northern Nigeria. OpenIDEO Report. 2014. https://challenges.openideo.com/challenge/zero-to-five/research/health-camps-expanding-access-to-healthcare-during-polio-immunization-campaigns-in-northern-nigeria. Accessed 12 January 2016.

[13] IRIN News (2012). Thousands at Risk of Polio in Somalia’s Port City of Kismayo.

[14] McGirk T. Taliban Assassins Target Pakistan’s Polio Vaccinators. National Geographic. 2015. http://news.nationalgeographic.com/2015/03/150303-polio-pakistan-islamic-state-refugees-vaccination-health/. Accessed 12 January 2016.

[15] Boone J. Bomb attack at polio vaccination centre kills 15 in Pakistani city of Quetta. The Guardian. 2016. http://www.theguardian.com/world/2016/jan/13/bomb-attack-at-polio-vaccination-centre-kills-14-in-pakistani-city-of-quetta. Accessed 14 January 2016.

[16] Islamic Advisory Group for Polio Eradication. http://www.iag-group.org/iag/home/. Accessed 18 January 2016.

[17] World Health Organization (2015). Introduction of inactivated polio vaccine and switch from trivalent to bivalent oral poliovirus vaccine worldwide, 2013–2016. Wkly Epidemiol Rec.

[18] World Health Organization. Emergencies, preparedness, response. Circulating vaccine-derived poliovirus – Ukraine. September 1, 2015. http://www.who.int/csr/don/01-september-2015-polio/en/. Accessed 1 March 2016.

[19] Independent Monitoring Board of the Global Polio Eradication Initiative. Now is the Time for Peak Performance. Twelfth Report: October 2015. http://www.polioeradication.org/Portals/0/Document/Aboutus/Governance/IMB/13IMBMeeting/13IMB_Report_EN.pdf.

[20] Asghar H, Diop OM, Weldegebriel G, Malik F, Shetty S, El Bassioni L (2014). Environmental surveillance for polioviruses in the Global Polio Eradication Initiative. J Infect Dis.

